# Neutropenic Enterocolitis as a Complication of Autologous Stem Cell Transplant in Patients With Multiple Myeloma: A Case Series

**DOI:** 10.7759/cureus.24475

**Published:** 2022-04-25

**Authors:** Nadia Belmoufid, Sanae Daghri, Soukaina Driouich, Anass Nadi, Nouama Bouanani

**Affiliations:** 1 Department of Hematology, Faculty of Medicine, Mohammed VI University of Health Sciences (UM6SS), Casablanca, MAR; 2 Department of Gastroenterology and Hepatology, Faculty of Medicine, Mohammed VI University of Health Sciences (UM6SS), Casablanca, MAR

**Keywords:** asct, autologous stem cell transplants, typhlitis, melphalan, multiple myeloma, autologous hematopoietic stem cell transplant, neutropenic colitis

## Abstract

Neutropenic enterocolitis (NE) is a rare but severe complication occurring in neutropenic patients undergoing intensive chemotherapy. Mortality is high, so early diagnosis is required to start urgent medical or surgical treatment. Data analysis of the development of NE after hematopoietic stem cell transplantation remains scarce. The aim of this case series is to discuss five out of 100 patients receiving autologous stem cell transplants (ASCTs) for multiple myeloma complicated with NE between 2016 and 2020 in the hematology department of the Cheikh Khalifa International University Hospital, Casablanca, Morocco. The patients were diagnosed with IgA and IgG multiple myeloma and aged between 58 to 64 years. They received induction therapy with four cycles of a triplet regimen including a proteasome inhibitor, an immunomodulatory drug, and corticosteroids, allowing a complete remission. Intensification was based on ASCT with melphalan at 200 mg/m2. The period of aplasia was marked by the sudden appearance of NE, diagnosed based on clinical, biological, and imaging criteria. Treatment included antibiotherapy and supportive care. We report no complications in our cases, nor the need for surgical care. Therefore, we consider that early diagnosis and treatment allowed a good evolution in our case series. The management of NE must be multidisciplinary associating hematologists, gastroenterologists, radiologists, and biologists. More studies and trials are needed to establish specific diagnostic criteria and better treatment options.

## Introduction

Neutropenic enterocolitis (NE) or typhlitis is a rare diagnostic and therapeutic emergency occurring primarily in neutropenic patients receiving intensive chemotherapy [1]. Mortality is high; therefore, early diagnosis based on clinical examination and imagery is required. Treatment is controversial, with possibilities ranging from conservative medical management to surgical intervention [1]. There are no randomized trials or high-quality cohort studies addressing important aspects of the diagnosis or management of the disease and scarce data analyzing the development of NE after hematopoietic stem cell transplantation. The aim of this case series is to discuss five cases of NE complicating autologous stem cell transplants (ASCT) between 2016 and 2020 in the hematology department of the Cheikh Khalifa International University Hospital, Casablanca, Morocco, as well as shed light on recent findings in the literature. 

## Case presentation

Out of a 100 patients diagnosed with IgA and IgG multiple myeloma, five patients aged 64, 64, 63, 61, and 58 years, respectively, received induction therapy with four cycles of bortezomib, thalidomide, and dexamethasone or four cycles of bortezomib, cyclophosphamide, and dexamethasone allowing a complete remission in all cases. Induction was followed by intensification using high dose melphalan at 200 mg/m2 followed by ASCT. All five cases presented during aplasia, with fever ranging from 38°C to 39°C, vomiting, abdominal pain, and hematochezia or gastrointestinal bleeding.

**Figure 1:**
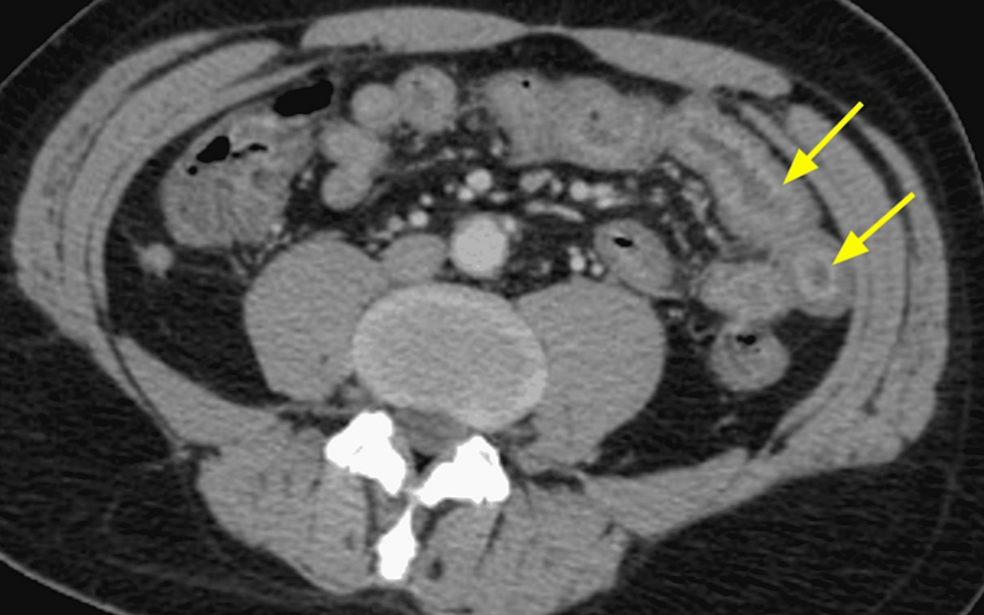
Contrast-enhanced CT image for Case 1 (axial plane) showing thickening of the colonic wall and increased mucosal enhancement (arrows)

**Figure 2:**
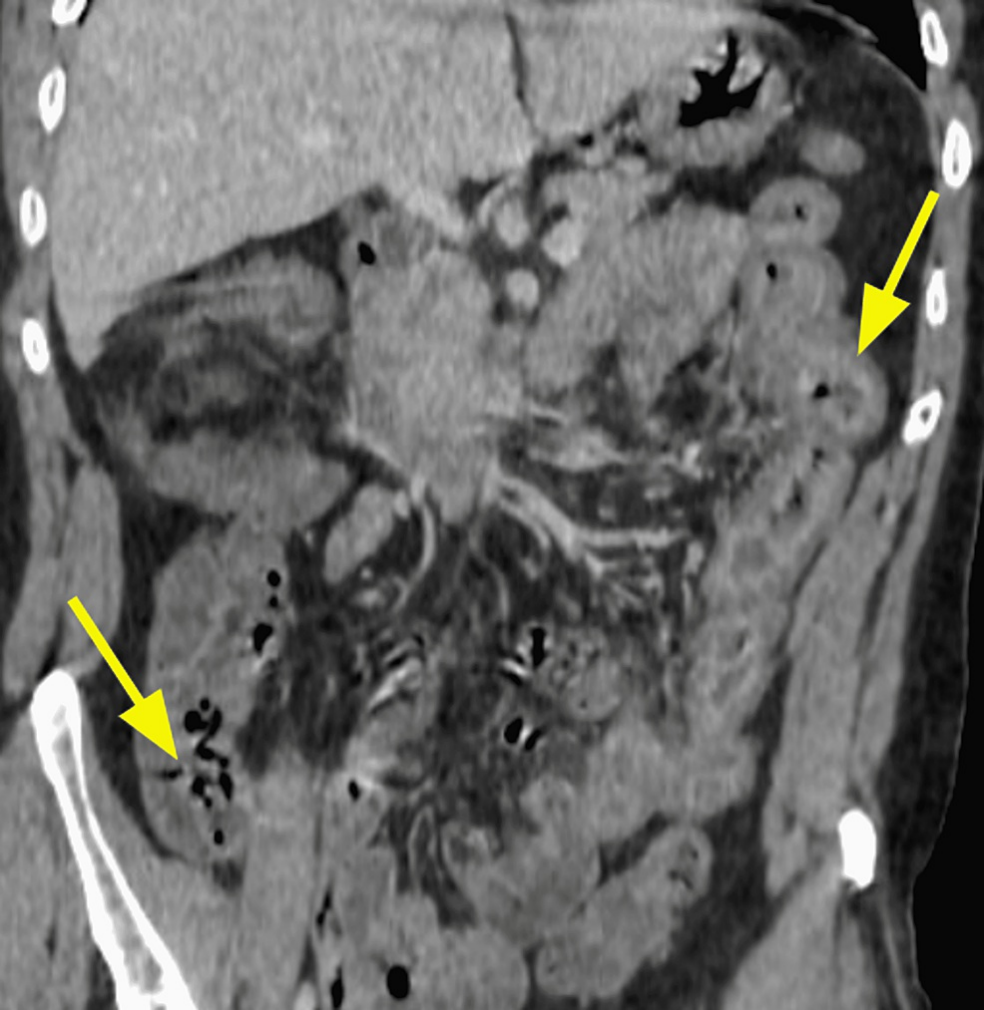
Contrast-enhanced CT image for Case 1 (coronal plane) demonstrating thickening of the caecum, the right and left large bowel wall, and increased mucosal enhancement (arrows)

**Figure 3:**
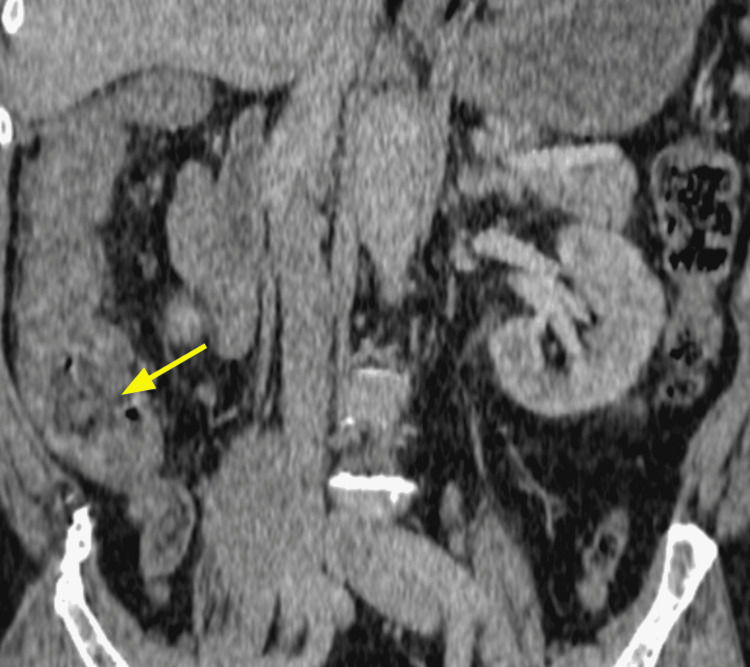
Contrast-enhanced CT for Case 2 (coronal plane) demonstrating large right bowel and thickening of the caecum wall with infiltration of the pericolic fat (arrow)

**Figure 4:**
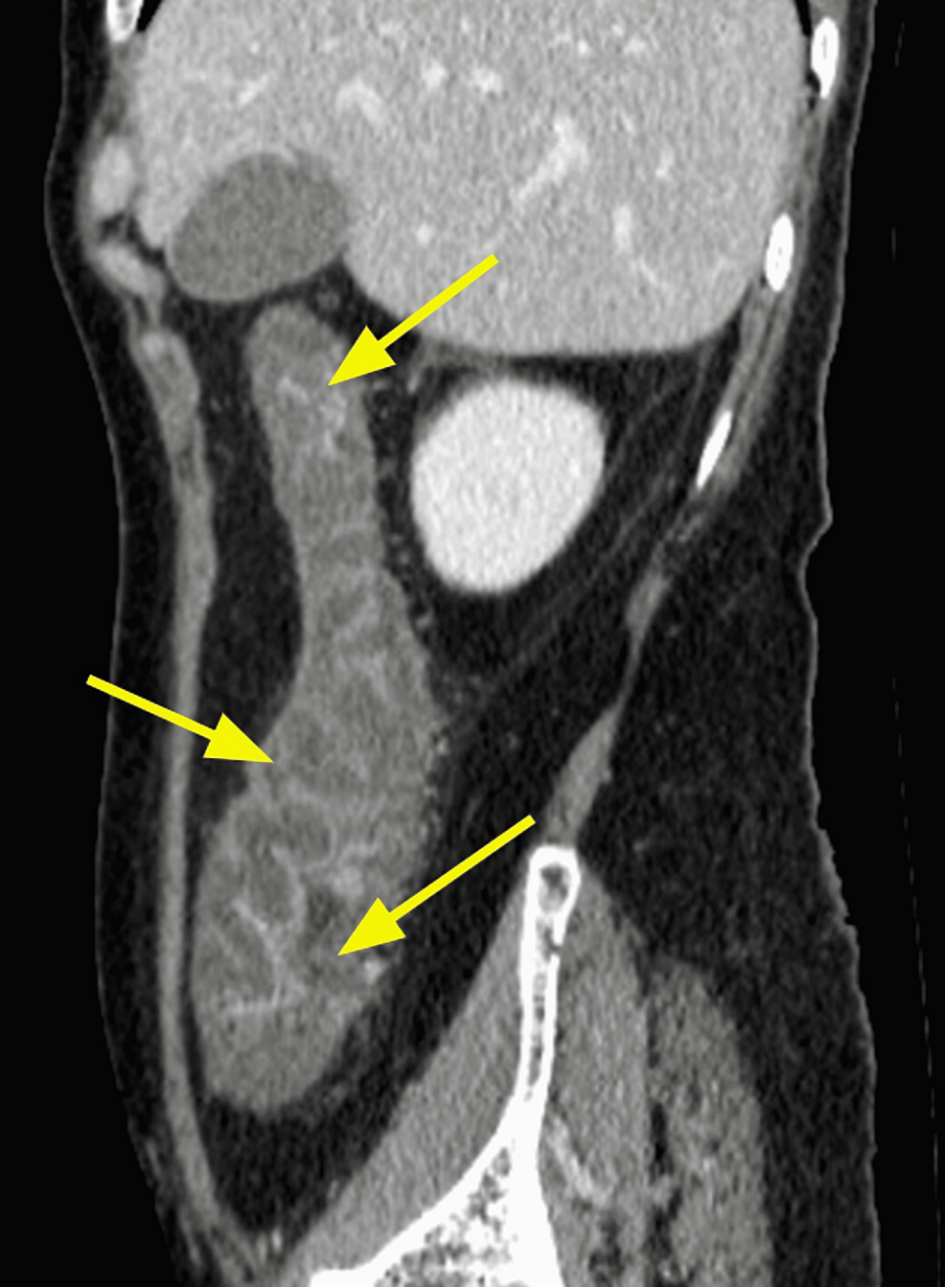
Contrast-enhanced CT for Case 3 (sagittal plane) demonstrating thickening of colonic wall and infiltration of the pericolic fat forming the "accordion sign" (arrows)

**Figure 5:**
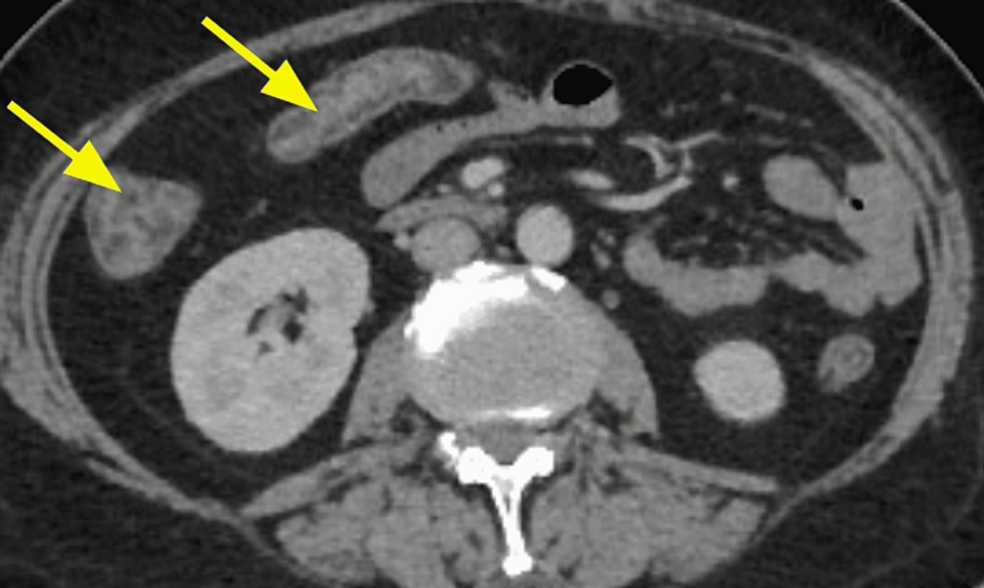
Contrast-enhanced CT image for Case 4 (axial plane) demonstrating thickening of the large bowel wall and increased mucosal enhancement (arrows)

**Figure 6:**
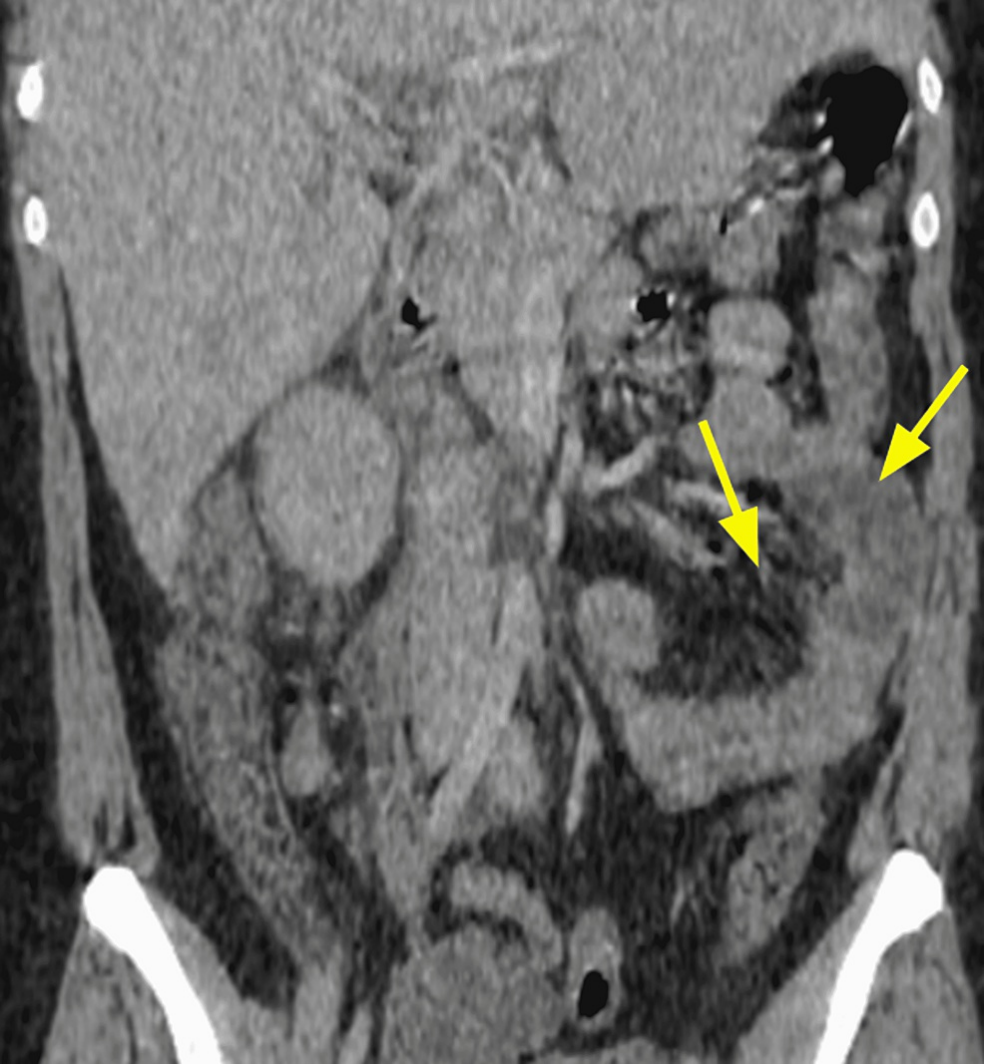
Contrast-enhanced CT image for Case 5 (coronal plane) showing thickening of left large bowel wall and infiltration of the pericolic fat (arrows)

The diagnosis was based on clinical, biological, and radiological criteria classified as major (neutropenia < 500 x 10^9/L, fever, thickening of the bowel wall > 4 mm) and minor criteria that are not specific (abdominal pain or cramp, diarrhea, digestive bleeding) according to Gorschlüter et al.'s research, established in 2005 [2]. Biological testing identified *Entamoeba histolytica* in one case on stool culture. *Clostridium difficile* was negative in all stool cultures. Bloodstream infections were identified in one case with *Escherichia coli* and with *Enterococcus faecium* in another case. Cytobacteriological examination of urine was negative in all cases. Abdominopelvic scans were performed on day two after the onset of the symptoms and identified thickening of the colonic wall without signs of complication in all five cases; infiltration of the pericolic fat was observed in some cases (Figures 1, 2, 3, 4, 5, 6).

Treatment was based on bowel rest, intravenous rehydration and parenteral alimentation, granulocytic colony-stimulating factors (G-CSF), and broad-spectrum antibiotics (ceftazidime and metronidazole) resulting in a good clinical and biological improvement within the first 48 hours of treatment. Ascension of white blood cells occurred between day 12 and day 13 of reinjection of stem cells. No complications such as perforation, septic shock, or ileus were observed. Clinical, biological, and imaging presentations of each patient are available in Table 1.

**Table 1:** Clinical, biological, and radiological presentation of the patients VTD: Velcade, thalidomide, dexamethasone; VCD: Velcade, cyclophosphamide, dexamethasone; MM: multiple myeloma; M: male; F: female; Hb: hemoglobin

	Case 1	Case 2	Case 3	Case 4	Case 5
Age	64	64	61	63	58
Sex	F	M	M	M	F
Comorbidities	Diabetes	-	-	-	-
Diagnosis	MM IgG Kappa	MM IgG Kappa	MM IgG Kappa	MM IgG Lambda	MM IgA Kappa
Chemotherapy	VTD	VTD	VCD	VTD	VTD
Radiotherapy	Yes				
Clinical presentation	Fever, mucositis, abdominal pain, diarrhea, hematochezia, or gastrointestinal bleeding	Fever, dysentery, vomiting	Fever, dysentery, diarrhea	Fever, dysentery, diarrhea	Fever, dysentery, diarrhea, hematochezia, or gastrointestinal bleeding
Hb (g/dl)	7	8	9	8	7
WBC count (10^9/L)	0.5	0.8	0.2	0.7	0.3
Neutrophil count (10^9/L)	0	0.03	0	0.02	0
Platelet count (10^9/L)	10	15	9	40	10
Stool culture	Entamoeba histolytica	-	-	-	-
Clostridium difficile	-	-	-	-	-
Bloodstream infection	-	Escherichia coli	-	Enterococcus faecium	-
Abdominal scan	Thickening of the colonic wall and increased mucosal enhancement (Figure 1, 2)	Thickening of the right colonic wall and caecum infiltration of pericolic fat (Figure 3)	Thickening of the colonic wall and infiltration of pericolic fat (Figure 4)	Thickening of the colonic wall and increased mucosal enhancement (Figure 6)	Thickening of the colonic wall infiltration of pericolic fat (Figure 6)

## Discussion

NE, also known as typhlitis (from Greek typhlon ("blind"), referring to the caecum), is an acute life-threatening condition manifesting in neutropenic patients as acute ulcerative colitis occurring 10 to 14 days after cytotoxic chemotherapy. It is classically characterized by transmural inflammation of the caecum and the appendix with involvement of the ascending colon and ileum in patients with severe myelosuppression. It was first described in pediatrics during the induction of leukemia [3]. The exact incidence of this manifestation is still unknown; a systematic review published in 2005 suggested an incidence of 5.6% in adults hospitalized for hematologic malignancies or under chemotherapy for solid tumors [2]. Other studies in the literature report it to vary between 2.8% and 5% in patients treated with intensive chemotherapy for both hematologic and solid malignancies [4]. The exact pathogenesis is not completely understood but several studies report the major role of intensive chemotherapy as it induces mucosal damage, epithelial cell apoptosis, increased mucosal permeability, and pro-inflammatory cytokines release [5], as well as other factors such as intramural hemorrhage due to thrombocythemia and neutropenia itself as it reduces the intestinal immune response to microbial invasion. Cytotoxic chemotherapy and/or radiotherapy are shown to cause microbiota imbalance that leads to mucosal damage, especially in patients under antibiotics or antifungal treatment [6]. These initial conditions lead to intestinal edema, engorged vessels, and a disrupted mucosal surface as well as a predisposition to infection and necrosis, thereby altering intestinal motility [1].

The clinical presentation of NE is mainly a triad of fever, diarrhea, and abdominal pain localized in the right iliac fossa that can become diffuse. It may also be complicated from the start with ascites, hemorrhage, peritonitis, or a septic shock as a result of necrosis or perforation, which constitutes a surgical emergency [6]. The diagnosis is perplexing because of the non-specific signs of NE. It can mimic several other pathologies such as pseudomembranous colitis, outbreaks of chronic inflammatory bowel disease, appendicitis, or ischemic colitis. A high index of suspicion is needed to avoid misdiagnosis [7]. Gorschlüter et al. established and published diagnostic criteria in 2005 based on major signs such as neutropenia, fever, and colonic thickening on the abdominal CT scan or ultrasound and minor criteria such as abdominal pain or abdominal cramp, abdominal distension, lower gastrointestinal bleeding, and diarrhea (Table 2) [2]

**Table 2:** Diagnosis criteria ANC: absolute neutrophil count Based on Gorschlüter et al., 2005 [2]

Type of criteria	Findings	Remarks
Major	Neutropenia, bowel wall thickening on CT scan or ultrasound exam, fever	ANC < 500 10^9 cells/L > 4 mm (transverse scan) thickening in any segment of the bowel for at least 30 mm length (longitudinal scan) > 38.3° (oral or rectal)
Minor/non-specific	Abdominal pain, abdominal distension, abdominal cramping, diarrhea, lower gastrointestinal bleeding	> 3 on visual analog scale (1-10)

Bedside ultrasound is a non-invasive and radiation-free imaging technique for early diagnosis of NE; it provides real-time images and is useful in identifying inflammation, abscesses, and bowel wall thickening, but it is limited as it cannot be used on obese patients and it can be impeded by gas bubbles [8]. The CT scan has many advantages when compared to ultrasound and the traditional contrast enema, which is why it is now accepted as the primary imaging modality for patients who present abdominal pain. A CT scan is capable of visualizing inflamed bowel and diverticula, pericolic fat stranding, and pericolic and colonic complications, which result in a more accurate diagnosis for the patient, along with a better standard of care [8,9].

The etiological approach is based on microbiological examinations, including blood culture and bacterial and parasitological examination of stools. Several studies reported the polymicrobial causative effect including gram-negative bacilli, gram-positive cocci, anaerobes, and fungi. The most commonly isolated organisms are *Pseudomonas aeruginosa*, *Escherichia coli*, *Klebsiella* spp, viridans group streptococci (VGS), enterococci, *Bacteroides* spp, *Clostridium* spp, and *Candida* spp [5,6]. Organisms such as *Clostridium septicum* and *Stenotrophomonas maltophilia* have been shown to correlate with a worse outcome and severe sepsis [10]. Melphalan is an alkylating agent that is known to cause gastrointestinal and oral mucositis, which represents a contributing factor to the installation of NE. The incidence of grade 3 or 4 gastrointestinal mucositis, according to the WHO, can be as high as 20-60% in patients undergoing hematopoietic stem cell transplantation depending on the intensity of the chemotherapy used [11]. A retrospective study identified 12% of cases of NE secondary to intensive chemotherapy in patients undergoing stem cell transplant of different hemopathies including Hodgkin's lymphomas, non-Hodgkin's lymphomas, multiple myeloma, and acute myeloid leukemia. NE was identified in one-third of the studied population and mostly in patients intensified with carmustine, etoposide, melphalan, and cytarabine (BEAM) compared to patients receiving intensification with melphalan alone in multiple myeloma. The severity of the clinical presentation and complications occurred mainly in patients undergoing BEAM regimen intensification in non-Hodgkin's lymphomas [6]. 

NE occurring after ASCT is complicated by perforation, digestive bleeding, pericæcal abscesses, and bacilli gram-negative sepsis in approximately 80% of cases. Therefore, NE should be suspected early as aggressive surgical or medical management may avoid a fatal outcome [9]. Mortality rates reported for NE are as high as 50%, especially if the patient has transmural inflammation or bowel perforation [12]. Older studies done in the 1980s reported mortality rates ranging from 50% to 100% while recent studies show a lower mortality rate of 30-50% due to early suspicion, diagnosis, and management [13,14].

Management of NE is based on medical and sometimes surgical treatment. Supportive care is essential and includes platelet transfusions, bowel rest with nasogastric suction, intravenous fluids, and parenteral nutrition if needed [15]. There are no trials to date to evaluate different treatment regimens but multiple case reports and studies showed the efficacy of antimicrobial therapy based on the patient’s antimicrobial exposure, bacteremia, and local resistance pattern. Anti gram-negative bacilli empirical antibiotherapy using piperacillin-tazobactam or carbapenem or antipseudomonal cephalosporin such as cefepime with metronidazole is indicated [16]. Anti gram-positive cocci antibiotics such as vancomycin are considered if mucositis is associated with the clinical presentation or if *Clostridium difficile *is isolated in the microbiological findings [17]. The guidelines do not address concrete treatment recommendations for fungal NE [12]. The use and benefit of G-CSF are still debated [18] but it represents one of the pillars in treating NE as shown in multiple case reports. Surgical intervention is indicated in complicated cases of NE such as perforation, pneumoperitoneum, and persistent gastrointestinal bleeding [19].

## Conclusions

Typhlitis or NE is a rare complication occurring during ASCT. The toxic role of anti-tumor agents and/or bacterial translocation has been described. High-dose melphalan causes oral and gastrointestinal mucositis contributing to the development of NE in immunosuppressed patients. The prognosis and mortality rates have evolved due to the early suspicion and diagnosis as well as adequate treatment. Management must be multidisciplinary associating hematologists, oncologists, gastroenterologists, radiologists, and biologists. Early diagnosis and treatment in the cases highlighted in our report allowed a good evolution without complications. More studies and trials are needed to establish diagnosis criteria and treatment indications.
